# The ALLEGRO trial: a placebo controlled randomised trial of intravenous lidocaine in accelerating gastrointestinal recovery after colorectal surgery

**DOI:** 10.1186/s13063-022-06021-5

**Published:** 2022-01-28

**Authors:** Hugh M. Paterson, Seonaidh Cotton, John Norrie, Susan Nimmo, Irwin Foo, Angie Balfour, Doug Speake, Graeme MacLennan, Andrew Stoddart, Karen Innes, Sarah Cameron, Lorna Aucott, Kirsty McCormack

**Affiliations:** 1 University of Edinburgh, Edinburgh, UK; 2 Centre for Healthcare Randomised Trials, University of Aberdeen, Aberdeen, UK; 3 Edinburgh Clinical Trials Unit, Usher Institute, University of Edinburgh, Edinburgh, UK; 4 Anaesthetics Department, Western General Hospital, Edinburgh, UK; 5 Colorectal Surgery, Western General Hospital, Edinburgh, UK

**Keywords:** Colorectal surgery, Minimally invasive surgery, Recovery, Pain, Analgesia, Gastrointestinal, Intravenous lidocaine, Ileus, RCT, Protocol

## Abstract

**Background:**

Return of gastrointestinal (GI) function is fundamental to patient recovery after colorectal surgery and is required before patients can be discharged from hospital safely. Up to 40% of patients suffer delayed return of GI function after colorectal surgery, causing nausea, vomiting and abdominal discomfort, resulting in longer hospital stay. Small, randomised studies have suggested perioperative intravenous (IV) lidocaine, which has analgesic and anti-inflammatory effects, may accelerate return of GI function after colorectal surgery. The ALLEGRO trial is a pragmatic effectiveness study to assess the benefit of perioperative IV lidocaine in improving return of GI function after elective minimally invasive (laparoscopic or robotic) colorectal surgery.

**Methods:**

United Kingdom (UK) multi-centre double blind placebo-controlled randomised controlled trial in 562 patients undergoing elective minimally invasive colorectal resection. IV lidocaine or placebo will be infused for 6–12 h commencing at the start of surgery as an adjunct to usual analgesic/anaesthetic technique. The primary outcome will be return of GI function.

**Discussion:**

A 6–12-h perioperative intravenous infusion of 2% lidocaine is a cheap addition to usual anaesthetic/analgesic practice in elective colorectal surgery with a low incidence of adverse side-effects. If successful in achieving quicker return of gut function for more patients, it would reduce the rate of postoperative ileus and reduce the duration of inpatient recovery, resulting in reduced pain and discomfort with faster recovery and discharge from hospital. Since colorectal surgery is a common procedure undertaken in every acute hospital in the UK, a reduced length of stay and reduced rate of postoperative ileus would accrue significant cost savings for the National Health Service (NHS).

**Trial registration:**

EudraCT Number 2017-003835-12; REC Number 17/WS/0210 the trial was prospectively registered (ISRCTN Number: ISRCTN52352431); date of registration 13 June 2018; date of enrolment of first participant 14 August 2018.

## Administrative information

Note: the numbers in curly brackets in this protocol refer to SPIRIT checklist item numbers. The order of the items has been modified to group similar items (see http://www.equator-network.org/reporting-guidelines/spirit-2013-statement-defining-standard-protocol-items-for-clinical-trials/).

**Table:** 

Title {1}	The ALLEGRO trial: A placebo controlled randomised trial of intravenous lidocaine in accelerating gastrointestinal recovery after colorectal surgery
Trial registration {2a and 2b}.	EudraCT Number 2017-003835-12 ISRCTN Number: ISRCTN52352431 Prospectively registered); date of registration 13 June 2018; date of enrolment of first participant 14 August 2018
Protocol version {3}	Version 5, 8 September 2020
Funding {4}	National Institute for Health Research (NIHR) Health Technology Assessment project no. 15/130/95
Author details {5a}	^1^Academic Coloproctology, University of Edinburgh, Western General Hospital, Edinburgh, UK; ^2^Centre for Healthcare Randomised Trials, University of Aberdeen, Aberdeen, UK; ^3^Edinburgh Clinical Trials Unit, University of Edinburgh, Edinburgh, UK; ^4^Anaesthetic Department, Western General Hospital, Edinburgh, UK; ^5^Usher Institute, University of Edinburgh, UK
Name and contact information for the trial sponsor {5b}	Co-sponsor 1: University of Edinburgh, ACCORD, The Queen’s Medical Research Institute, 47 Little France Crescent, Edinburgh EH16 4TJ. Email enquiries@accord.scot Co-sponsor 2: NHS Lothian, ACCORD, The Queen’s Medical Research Institute, 47 Little France Crescent, Edinburgh EH16 4TJ. Email enquiries@accord.scot
Role of sponsor {5c}	The sponsor played no part in study design; and will play no part in the collection, management, analysis, and interpretation of data; writing of the report; and the decision to submit the report for publication.

## Introduction

### Background and rationale {6a}

Approximately 30,000 patients per year undergo colonic surgery in the UK. Discharge from hospital after colectomy is inextricably linked to recovery of gastrointestinal (GI) function. Surgery to remove part of the colon and/or rectum causes unavoidable interruption of normal gut propulsive contraction, and almost no patients are discharged from hospital before GI function recovers. With modern care, most patients undergoing laparoscopic colectomy are independently mobile, requiring no or minimal analgesia, and free from intravenous fluids and invasive monitoring (e.g. urinary catheters) within 48–72 h. However, less than half will have regained GI function by this time point, and 10–20% will go on to suffer substantial delay to return of GI function (aka postoperative ileus) characterised by nausea, vomiting, complete constipation and abdominal distension. This pathophysiology is complex and involves interaction of endogenous factors (including pain pathways, host endocrine- and inflammation-mediated stress responses, exaggerated sympathetic autonomic activity and endogenous opioids) and exogenous factors (bowel handling during surgery, opioid analgesics, anaesthetic agents, immobility). Usually self-limiting, it prolongs hospital stay by a few days in most cases but can last up to 10 days if severe [1]. Affected patients cannot tolerate oral intake due to nausea and vomiting and require inpatient support with intravenous fluids and ongoing opioid analgesia. Although GI transit is absent, digestive fluid continues to be secreted but not absorbed (bile, gastric acid, pancreatic enzymes) causing vomiting, dehydration and progressive abdominal distension. Many patients require decompression of the fluid-distended gut by insertion of a nasogastric tube, a very uncomfortable experience that some patients rate the worst part of their postoperative recovery. Furthermore, in an increasingly elderly and frail population, postoperative ileus can contribute to development of other major complications including pulmonary aspiration, pneumonia and acute kidney impairment [2–4]. In the previous era of open colorectal surgery, many surgeons regarded delayed return of gut function as a “normal” part of a prolonged post-operative recovery. Accordingly, the true prevalence was seldom recorded; best estimates suggest it affected up to 40% of cases. However, in the last decade, minimally invasive surgical techniques (laparoscopic and robotic “key-hole” surgery) and evidence-based “Enhanced Recovery” perioperative management care pathways have reduced average length of hospital stay from 9 days to 4–6 days and have of themselves reduced the prevalence of delayed return of gut function. The success of these techniques in accelerating recovery has highlighted that delayed return of GI function has become the main barrier to discharge for a significant proportion of minimally invasive colectomy patients.

There is no specific therapy for postoperative ileus; treatment is supportive until GI function returns spontaneously. Alvimopan (Entereg®) is a selective μ-opioid receptor antagonist evaluated in a number of North American randomised controlled trials (RCTs) where it has been shown to be of benefit in reducing incidence of postoperative ileus [5]. However, it has not gained widespread use due to cost and concerns regarding its cardiac side-effect profile and is not licensed for use in the UK.

Lidocaine, familiar as a local anaesthetic, has analgesic and anti-inflammatory effects when administered systemically by intravenous (IV) infusion and hence has been repurposed as an anaesthetic adjunct in abdominal surgery. Studies in various surgical procedures have shown reduced early postoperative pain scores and reduced opioid analgesia requirements. There also seems to be a consistent benefit on measures of GI function recovery, including time to first flatus, time to first bowel movement, postoperative nausea and risk of postoperative ileus. However, the limitation of meta-analysis in this area is the considerable heterogeneity of studies included: a variety of surgical procedures, a mix of open and laparoscopic techniques, inconsistent lidocaine dose/treatment duration, inconsistent perioperative management protocols and study endpoints [6–8]. There are only three published RCTs of intravenous lidocaine in laparoscopic colectomy, comprising a total of 181 patients. Two were conducted in Europe and reported length of stay consistent with modern NHS practice (3 to 5 days) [9, 10]. Both studies reported reduced analgesic requirements, faster return of gut function and a 1-day reduction in median length of stay. The third study was conducted in South Korea and found a trend towards faster GI recovery but no statistically significant benefit. However, perioperative management practices were not consistent with NHS norms and the excessive median length of stay of 9 days makes this study difficult to interpret [11].

There is some controversy in the use of perioperative IV lidocaine. Although it has many advocates in the anaesthetic community, there is a risk of fatal toxicity in overdose. The dose and duration of IV lidocaine used in ALLEGRO is at the lower threshold of the known therapeutic range; the greatest risk of toxicity is from human error in drug administration. Although guidelines for use of IV lidocaine were published recently (subsequent to the start of the ALLEGRO study), an accompanying editorial was not supportive [12, 13]. As emphasised in the ensuing journal correspondence, there is scant randomised evidence from large trials, and ALLEGRO is the only adequately powered trial of IV lidocaine currently recruiting [14].

Intravenous lidocaine is cheap, familiar and easy to administer within existing NHS perioperative practice. If effective in improving return of GI function postoperatively it would achieve faster recovery and shorter hospital stay for patients undergoing colorectal surgery, resulting in a major cost saving for the NHS from a common (and resource-intensive) surgical intervention.

### Objectives {7}

#### Primary objective

The aim of the study is to measure whether perioperative intravenous lidocaine achieves faster return of GI function for more patients after laparoscopic colectomy. The primary objective is to compare the proportion of participants randomised to IV lidocaine or placebo that have achieved return of gut function at 72 h post-operatively (GI-3 recovery endpoint).

#### Secondary objectives

The secondary objectives are to compare the proportion of participants randomised to IV lidocaine or placebo in respect of:

- GI-2 recovery endpoint
- The rate of prolonged postoperative ileus
- Postoperative nausea and vomiting
- Analgesia requirements
- Quality of postoperative recovery using multi-dimensional patient-reported outcome tools and quality of life tools.

Additional secondary objectives include assessing whether perioperative intravenous lidocaine during colorectal surgery is cost-effective relative to current standard of care (colorectal surgery without intravenous lidocaine) and measuring the impact of variation in peri-operative care from Enhanced Recovery After Surgery guidelines.

The tertiary objectives are to compare the proportion of participants randomised to IV lidocaine or placebo in respect of:

- Total length of stay
- 30- and 90-day mortality
- Unplanned re-admissions within 30 days of hospital discharge
- Reoperation/major complications (Clavien-Dindo classification grade 3 and above) [15]
- Qualitative analysis of recovery beyond hospital (General Practitioner (GP) visits, district nurse visits).

### Trial design {eight}

The trial design is as follows: parallel group effectiveness trial of perioperative IV lidocaine infusion versus placebo (0.9% NaCl) with 1:1 allocation.

## Methods: participants, interventions and outcomes

### Study setting {9}

We are recruiting patients in 25–30 colorectal surgery units across the UK. A list of study sites can be obtained from the trial website (https://w3.abdn.ac.uk/hsru/ALLEGRO/Public/Public/index.cshtml) and will be included in the final report from the study.

### Eligibility criteria {10}

#### Inclusion criteria

Patients scheduled for elective minimally invasive (laparoscopic or robotic) colorectal resection for colorectal cancer, benign polyps, benign stricture or diverticular disease at participating UK colorectal surgical units. Right hemicolectomy, extended right hemicolectomy, left colectomy, sigmoid colectomy, subtotal colectomy with ileosigmoid or ileorectal anastomosis and high anterior resection are eligible.

#### Exclusion criteria

- Planned epidural anaesthesia
- Planned regional or local continuous infusion of lidocaine at the same time as lidocaine infusion
- Planned open surgery
- Current pregnancy
- Breastfeeding
- Age < 18 years
- Patients lacking capacity to give informed consent
- Known or suspected allergy to lidocaine or amide-type local anaesthetics
- Current complete heart block
- Current severe liver dysfunction (Child’s A or greater)
- Current renal failure (estimated glomerular filtration rate (eGFR) < 30)
- Patients participating in the active intervention phase of another therapeutic clinical trial (or other interventional trial) unless a co-enrolment agreement is in place
- Patients having surgery for indications other than those noted above
- Rectal cancer below the peritoneal reflection in which total mesorectal excision is planned
- Rectal cancer patients who have received any neoadjuvant radiotherapy
- A preoperative surgical plan to form any new stoma during the primary procedure

### Who will take informed consent? {26a}

Consent from potential trial participants will be sought by a member of the local trial team trained in Good Clinical Practice (GCP). This will usually be in a face-to-face setting, during an out-patient appointment, pre-operative assessment or when the participant is admitted to hospital for their surgery. As part of the COVID-19 contingencies, if face-to-face consent is not possible, participants can complete the consent form at home and either post it back to the research team or bring it into hospital with them when they are admitted for surgery.

### Additional consent provisions for collection and use of participant data and biological specimens {26b}

As the most common indication for colectomy will be colorectal cancer, consent includes permission for storage of data to permit subsequent record linkage to analyse cancer-specific outcomes. Consent for this is sought at the time of consent to the main study and is optional.

### Interventions

#### Explanation for the choice of comparators {6b}

Intravenous lidocaine used in this setting is an adjunct to usual anaesthetic and perioperative analgesic practice. There is no alternative medication against which to test the intervention; hence, the comparator is a placebo of physiological (isotonic) 0.9% sodium chloride (NaCl) which looks identical to lidocaine.

#### Intervention description {11a}

In the intervention arm, an intravenous bolus of 2% lidocaine at induction of anaesthesia (1.5 mg/kg ideal body weight) infused over 20 min is followed by intravenous infusion of 1.5 mg/kg/h ideal body weight with a maximum rate of 120 mg/h for a minimum of 6 h up to a maximum of 12 h. Ideal body weight rather than actual body weight is used to prevent the possibility of toxicity by exceeding the upper therapeutic threshold of lidocaine in very overweight patients.

The bolus and intravenous infusion rate of the placebo are the same as the investigational medicinal product (IMP).

Continuous cardiac monitoring during the period of IMP/placebo infusion is required as systemic toxicity can provoke cardiac arrhythmias.

The planned duration of infusion will be determined pre-operatively by the participating unit’s normal postoperative availability of continuous cardiac monitoring. Units where normal postoperative disposal is to the standard inpatient ward, where facility for cardiac monitoring is not available, will aim to administer the infusion for a minimum of 6 h (operating time plus theatre recovery suite time). Before the patient is moved to the ward, the infusion will be stopped. This means that (i) if the operating time plus theatre recovery suite time is less than 6 h, the duration of the infusion will also be less than 6 h, and (ii) if the operating time plus theatre recovery time is 6 h or more, the duration of the infusion will be more than 6 h but will be stopped before 12 h.

Units where normal postoperative disposal is to HDU, or other clinical area where cardiac monitoring is available, will aim to administer the infusion for up to 12 h. If the patient is moved to a ward without cardiac monitoring facilities before the 12-h period has been completed, the infusion will be stopped.

#### Criteria for discontinuing or modifying allocated interventions {11b}

As noted above (in the “Intervention description {11a}” section), the infusion will run for a maximum of 12 h but will be stopped if continuous cardiac monitoring is stopped before the end of the planned duration. Infusion of lidocaine will be stopped immediately if the patient has symptoms of acute systemic toxicity. The treatment of systemic lidocaine toxicity is supportive treatment and lipid rescue.

#### Strategies to improve adherence to interventions {11c}

The IMP/placebo infusion will be monitored as a part of standard nursing care for patients. As the IMP/placebo is infused for a maximum of 12 h, all participants will be inpatients throughout the infusion. Infusion rate tables have been provided to local study teams to minimise user error in administrating the IMP to participants.

#### Relevant concomitant care permitted or prohibited during the trial {11d}

Concomitant continuous infusions of local anaesthetic agents are prohibited to prevent cumulative systemic toxicity; hence, planned epidurals or continuous infusion of local anaesthetic via wound catheters are both exclusion criteria.

#### Provisions for post-trial care {30}

This is a one-off infusion given to participants during surgery and post-operatively. Usual care for participants continues throughout the surgery and post-operative period, during their recovery and after discharge.

### Outcomes {12}

Return of GI function after surgery is awkward to define. A variety of endpoints are reported in studies (nausea scores, time to passage of flatus, time to passage of stool, time to normal oral intake, etc.). Previous studies of return of GI function after colorectal surgery described and validated composite clinical endpoints (GI-3 and GI-2) and were validated by the authors in a separate feasibility study prior to ALLEGRO [1, 2, 16].

#### Primary outcome

The primary outcome is achievement (yes/no) of GI-3 recovery (a composite endpoint defined as the achievement of both of the following two events: tolerating diet without significant nausea or vomiting for three consecutive meals; AND passage of flatus OR stool) at 72 h after the start of operation.

#### Secondary outcomes

- Time to GI-3 recovery
- Time to GI-2 recovery (a composite endpoint defined as time from surgery to the time to establish both of the following two events: tolerating diet without significant nausea or vomiting for three consecutive meals; AND first passage of stool).
- Rate of prolonged postoperative ileus (failure to establish GI-3 by 120 h after surgery)
- Nausea and vomiting score
- Overall Benefit of Analgesia (OBAS) score
- Total postoperative opioid consumption
- Quality of recovery (QoR-15 patient-reported outcome measurement (PROM))
- EQ-5D-5L quality of life assessment
- Enhanced Recovery After Surgery (ERAS) protocol compliance
- Analysis of cost of care derived from hospital records, recorded GP visits and participant reported data up to 90 days
- Cost per quality-adjusted life years (QALYs) as observed over the 90 day trial period, and as modelled over longer term
- Total length of hospital stay (primary admission plus any readmission(s))
- Mortality at 30 and 90 days
- Major complications
- Record linkage analysis of survival (cancer-specific outcome data in appropriate patients up to 10 years)

### Participant timeline {13}

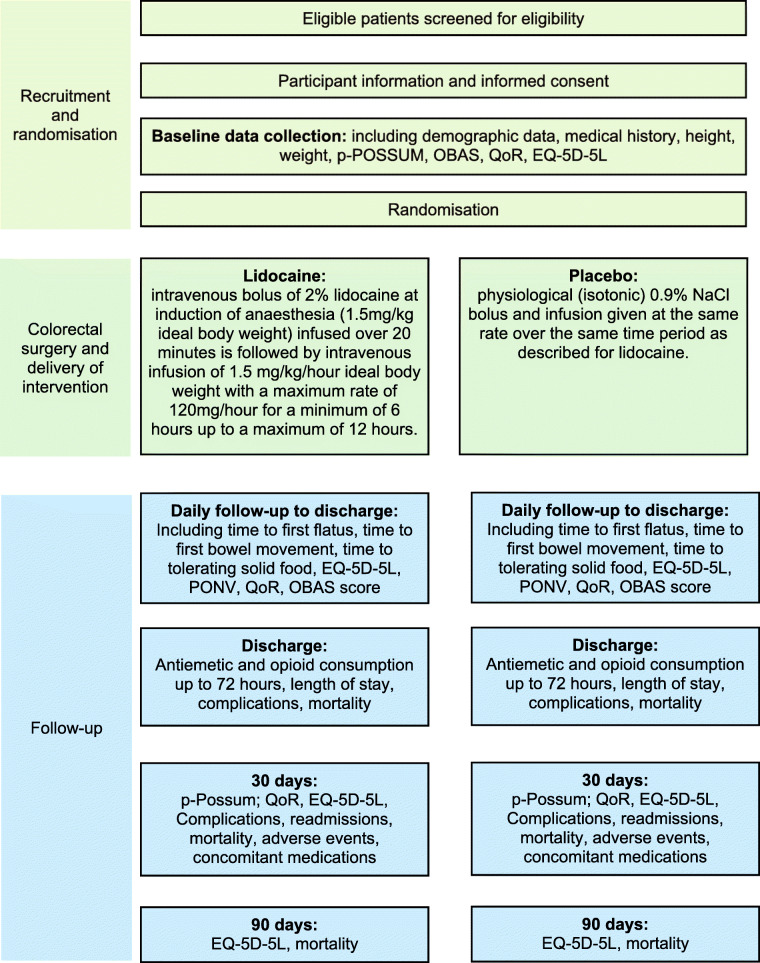



### Sample size {14}

In a feasibility study in laparoscopic colectomy conducted under real-world NHS conditions with NHS patients typical of the recruitment cohort proposed in this application, 22 of a total of 50 patients (44%) failed to achieve return of GI function (GI-3 endpoint) by the third postoperative day [2]. Ten patients (20%) went on to develop prolonged postoperative ileus (PPOI). Interpretation of data from other healthcare systems is made complex by reporting of various univariate endpoints, but control arm data support this event rate. Effect size in published reports ranges from 15 to 40% relative reduction in GI dysfunction rate, again using a variety of univariate endpoints.

Therefore, with a sample size of 562 randomised 1:1 to IV lidocaine or placebo the study will have 90% power at a two-sided 5% level of significance to detect a relative reduction of 33% from 40 to 26.8% (absolute reduction of 13.2%) in non-return of gut function at 3 days post-operatively (or if the event rate is lower, the same power to detect a 40% relative reduction from 30% to 18% (a 12% absolute reduction)). Given the primary endpoint is measured in hospital, there should be no appreciable loss to follow-up (the perioperative death rate is ~ 1% for the whole duration of hospital stay and the very few discharged before 3 days can be assumed to have achieved return of GI function). This size of study would also give similar power to detect a difference of 10% (from 20% to 10%) in the important secondary outcome of PPOI.

### Recruitment {15}

Eligible individuals are identified by their usual clinical team from planned elective operating lists and given a patient information sheet. Eligibility is confirmed, consent received, baseline details collected, and randomisation performed pre-operatively, where possible, at routine hospital visits.

As noted above (in the “Who will take informed consent? {26a}” section) where a face-to-face visit is not possible to carry out the recruitment procedures, participants can complete the consent form at home and either post it back to the research team or bring it into hospital with them when they are admitted for surgery. In such cases, eligibility is confirmed, baseline details collected, and randomisation performed pre-operatively.

### Assignment of interventions: allocation

#### Sequence generation {16a}

Following consent, a minimisation algorithm is used to randomly allocate participants (on a 1:1 basis) to receive intravenous lidocaine or placebo. This is available using a web-based application (available 24 h a day). The minimisation criteria are age (< 50 years, 50–74 years, 75 years and older), gender and trial centre.

#### Concealment mechanism {16b}

The web-based randomisation system ensures allocation concealment.

#### Implementation {16c}

The allocation sequence generation is embedded in the trial web site. Research nurses based at sites enrol participants and randomise them using the web-based randomisation system.

### Assignment of interventions: blinding

#### Who will be blinded {17a}

Participants, medical staff, local pharmacy staff and study staff/outcome assessors are all blinded in this study. Both study drug and placebo are clear colourless liquids packaged in identical containers by the manufacturer. At randomisation, the participant will be allocated a unique participant study number and assigned a numbered participant study drug pack. Randomisation also triggers a notification email to the local clinical trials pharmacy to provide the numbered pack to the local study team on receipt of a signed clinical trials prescription.

#### Procedure for unblinding if needed {17b}

In the event of the characteristic symptoms of lidocaine toxicity in a trial patient, it is appropriate to stop the infusion and treat as per current Association of Anaesthetists of Great Britain and Ireland guidelines without having to wait for code breaks/permissions etc. If necessary, the investigator can unblind a participant’s treatment allocation via the trial database or telephone. All investigators receive training on this prior to the site opening to recruitment.

### Data collection and management

#### Plans for assessment and collection of outcomes {18a}

Data are collected at baseline and at 1, 2, 3, 4, 5, 6 and 7 days after surgery, at discharge from hospital and at 30 and 90 days after surgery. The schedule for data collection is summarised in Table 1.

**Table 1:** Schedule for data collection

Assessment	Recruitment/baseline	POD 1	POD 2	POD 3	Daily from POD 4 until discharge	POD 7	Discharge	30 days	90 days
Assessment of eligibility criteria	✓								
Written informed consent	✓								
Pregnancy test (where applicable)									
Demographic data, contact details	✓								
Clinical history/past medical history	✓								
Drug history esp. laxatives	✓								
Height	✓								
Weight	✓	✓							
p-POSSUM	✓	✓ (part)						✓ (part)	
Operation type		✓							
Duration of operation		✓							
Blood loss (ml)^a^		✓							
OBAS score	✓	✓	✓	✓	✓	✓			
QoR score	✓	✓	✓	✓	✓	✓		✓	
PONV score		✓	✓	✓	✓				
EQ-5D-5L	✓	✓	✓	✓	✓	✓		✓	✓
CRP^b^	✓		✓	✓					
Time to first flatus			✓	✓	✓				
Time to first bowel movement			✓	✓	✓				
Time to tolerating solid food			✓	✓	✓				
antiemetic dose total up to 72 h							✓		
Total number of episodes vomiting		✓	✓	✓					
Total opioid consumption in-hospital up to 72 h							✓		
ERAS protocol compliance		✓	✓	✓					
Achievement of medical criteria for discharge Y/N				✓	✓				
Patient-reported readiness for discharge				✓	✓				
Length of stay (days)							✓		
Complications							✓	✓	
Unplanned readmissions								✓	
Mortality							✓	✓	✓
Adverse events		✓	✓	✓	✓	✓		✓	
Concomitant medications	✓	✓	✓	✓	✓	✓		✓	

^a^Millilitres

^b^C-reactive protein

#### Plans to promote participant retention and complete follow-up {18b}

Data are collected at routine clinic/hospital appointments and in hospital during postoperative recovery. It is highly unlikely that any patient will be discharged from hospital prior to achieving the primary endpoint; in such cases, this will be collected by phone at 7 days. For the participant reported outcomes collected (by telephone) at 7, 30 and 90 days after surgery, there is an acceptable visit window within which this data can be collected. We will aim to collect outcome data on all participants randomised, regardless of whether or not they received the intravenous lidocaine/placebo. There are no additional plans to enhance retention in ALLEGRO.

#### Data management {19}

All data are captured onto a paper-based case report form and entered by site staff onto a web-based case report form. All data are held securely on a server at the University of Aberdeen. The central trials team monitor data entry and ensure that missing or erroneous data are addressed as soon as possible after detection.

#### Confidentiality {27}

Data are stored in accordance with GCP and with the UK Data Protection Acts 1998 and 2018.

Unique participant identifiers (study number) are used to identify completed case-report forms and questionnaires. Records are kept in a secure storage area with limited access.

The Trial Website is secure sockets layer (SSL) secured, ensuring links between server and browser client are always encrypted. Access to patient identifiable information is limited to key personnel only and has appropriate user level access across a secure network, personal identifiable data held in the database will be encrypted to advanced encryption standard AES_256.

#### Plans for collection, laboratory evaluation and storage of biological specimens for genetic or molecular analysis in this trial/future use {33}

Not applicable—there are no biological specimens within this study.

### Statistical methods

#### Statistical methods for primary and secondary outcomes {20a}

Analysis will be by intention to treat. All baseline and outcome data will be described using appropriate summary statistics, e.g. frequency and proportions for dichotomous data, mean and standard deviation for continuous data (or median and interquartile range if more appropriate), and graphical methods. The primary outcome will be analysed using a generalised linear model with a logit link function and including minimisation covariates. Secondary outcomes will be analysed in a similar manner using a statistical model appropriate for the outcome (Cox regression for time-to-event outcomes, linear regression for continuous outcomes). All treatment effects will be presented with 95% confidence intervals. The planned analysis will be documented in the statistical analysis plan prior to analysis starting.

Full details of the Health Economic analysis will be specified in a Health Economic Analysis Plan prior to analysis [17]. Two complimentary forms of analysis will be undertaken: (1) a within trial analysis utilising self-reported EQ-5D-5L data, and health care resource utilisation (HCRU) extracted from hospital records detailed in Table 1 [schedule for data collection in the “Plans for assessment and collection of outcomes {18a}” section], undertaken via generalised linear modelling and a recycled predictions approach [18] to account for skew, and (2) simplified decision analytic modelling [19] incorporating any available wider literature [6] for longer term outcomes and low frequency high severity events.

To maximise UK policy relevance, both health economic analysis will follow National Institute for Health and Care Excellence (NICE) reference case recommendations [20] including adoption of an NHS and personal social service (PSS) costing perspective for primary analyses; application of standard UK price weights to HCRU to estimate cost [21, 22]; a cost-utility approach (results presented in terms of incremental cost per quality-adjusted life year (QALY), with QALYs derived from EQ-5D-5L); discount rate of 3.5% for both costs and QALYs (if applicable); use of probabilistic sensitivity analysis (PSA) to generate cost-effectiveness acceptability curves [18, 19] and deterministic sensitivity analysis on key assumptions where PSA is inappropriate.

#### Interim analyses {21b}

There are no planned interim analyses.

#### Methods for additional analyses (e.g. subgroup analyses) {20b}

We will explore whether outcomes are different between groups for minimisation variables (age, gender, site) and for type of surgery (right v left side colectomy) and by enhanced recovery compliance indicators. Additional planned sub-group analysis will be documented in the statistical analysis plan (SAP) or defined as post hoc analyses.

#### Methods in analysis to handle protocol non-adherence and any statistical methods to handle missing data {20c}

We do not anticipate any missing data due to the short timeframe of the primary outcome. However, should we observe substantial missing data, we will use multiple imputation and pattern mixture methods to explore the robustness of treatment effect estimates.

#### Plans to give access to the full protocol, participant level-data and statistical code {31c}

The full protocol is available as a supplement. Non-identifiable participant-level data may be available on request to the chief investigator (CI), Mr Hugh Paterson (hugh.paterson@ed.ac.uk).

### Oversight and monitoring

#### Composition of the coordinating centre and trial steering committee {5d}

The immediate trial team (CI, trial manager, data coordinator) meets every fortnight. On a monthly basis, the immediate team is joined by the wider trial team (statistician, health economist). A project management group (PMG) and trial steering committee (TSC) oversee the project. The PMG meets every 3 months and comprises the CI, grant holders and the trial office staff.

The TSC meets approximately every 6 months and includes an independent chair, clinical and methodological expertise and lay representative. Members of the PMG also attend the meetings of the TSC. Roles of the TSC are documented in the TSC charter, which all independent members have signed up to.

#### Composition of the data monitoring committee, its role and reporting structure {21a}

The data monitoring committee (DMC) meets approximately every 6 months to oversee the safety of participants in the trial. It includes an independent chair and independent members with clinical and methodological expertise. The CI, trial manager and other members of the PMG may attend the open session of the DMC. The DMC reports to the chair of the TSC. Roles are documented in the DMC charter, which all independent members have signed up to.

#### Adverse event reporting and harms {22}

Adverse events (AEs) will be identified and recorded from the time a participant signs the consent form to take part in the study until the 30th post-operative day. They will be asked about AEs daily during their admission, and during the 7- and 30-day telephone calls. When an AE occurs, it is the responsibility of the investigator, or another suitably qualified physician in the research team to review documentation related to the event. Relevant information will be recorded on the AE log and a serious adverse event (SAE) form if required—the specific requirements for recording events in ALLEGRO are described in Table 2 below.

**Table 2:** Event recording

Event	If the event is thought to be an adverse reaction	If the event is not thought to be an adverse reaction
Lidocaine toxicity within 2 h of infusion	Record on adverse event log (SAR^a^ if meets criteria for serious)	n/a
Transient events common after surgery (listed in the protocol as: pyrexia; anorexia, nausea, vomiting, constipation, pain; raised inflammatory markers; minor wound infections; dizziness; transient biochemical derangement e.g. hypokalaemia, hyponatraemia, hypomagnesaemia, hypophosphataemia, hypocalcaemia; transient self-limiting confusional state)	Record on adverse event log	Recorded on adverse event log
Complications of surgery Clavien-Dindo grade 1/2	Record on adverse event log	Recorded on adverse event log
Complications of surgery Clavien-Dindo grade 3+	Record as SAR/SUSAR; report to sponsor within 24 h of becoming aware	Record as outcome data on CRF^b^
Prolongation of admission for social reasons	n/a	Record as outcome data on CRF
Death	Record as SAR/SUSAR; report to sponsor within 24 h of becoming aware	Record as SAE; report to sponsor within 24 h of becoming aware

^a^Serious adverse reaction

^b^Case report form

The investigator (or delegate) is responsible for assessing each AE using standard definitions for severity, seriousness, causality (relatedness to the IMP) and (if the event is assessed as related to the IMP) whether it is expected or unexpected based on information contained within the Summary of Product Characteristics for lidocaine. The sponsor is responsible for notifying the regulatory authority and ethics committee of suspected unexpected serious adverse reactions SUSARs within defined timelines.

#### Frequency and plans for auditing trial conduct {23}

The trial office monitors aspects of the study on an ongoing basis as described in the study monitoring plan. The trial is monitored and audited by the sponsor. Individual sites may be monitored by their local NHS Research and Development (R&D) departments.

#### Plans for communicating important protocol amendments to relevant parties (e.g. trial participants, ethical committees) {25}

Changes to the protocol require the trial office to seek permission from the funder, sponsor, REC, Medicines and Healthcare products Regulatory Authority (MHRA, if appropriate) and NHS R&D offices.

Any changes in research activity, except those necessary to remove an apparent, immediate hazard to the participant in the case of an urgent safety measure, must be reviewed and approved by the chief investigator.

Proposed amendments will be submitted to the sponsor for classification and authorisation.

Amendments to the protocol will be submitted in writing to the appropriate REC, Regulatory Authority (MHRA where appropriate) and local R&D for approval prior to implementation.

Any amendments concerning modification of the intended use of the IMP or placebo will be notified to the manufacturer of IMP/placebo (Tayside Pharmaceuticals) for review and comment.

Following approval, Academic and Clinical Central Office for Research & Development (ACCORD) will provide Tayside Pharmaceuticals with a copy of the amended protocol and associated documents.

#### Dissemination plans {31a}

We will develop a publication and dissemination plan to include conference presentation(s) and journal publication(s). We plan to write to all participants to inform them of the trial results. We will also plan dissemination to relevant patient and clinical interest groups.

The results of the study, together with other mandated information, will be uploaded to the European clinical trials database within 1 year of the end of the study.

The results of the study will be disseminated to colorectal surgeons, anaesthetists and patient groups via the Association of Coloproctology and Great Britain and Ireland (ACPGBI), UK Perioperative Medicine Clinical Trials Network (POMCTN) and the Bowel Disease Research Foundation (BDRF) respectively.

## Discussion

Colorectal surgery is a core part of general surgery and undertaken in every acute NHS hospital from small district generals to university teaching institutions. Approximately 30,000 colectomies per year are undertaken in the UK alone; hence, a large number of patients could stand to benefit from an intervention that improves functional GI recovery. Data from small trials suggest a beneficial effect from intravenous perioperative lidocaine on this key determinant of recovery from colorectal surgery. ALLEGRO aims to be the definitive trial of perioperative IV lidocaine infusion in colorectal surgery within an NHS setting.

There is variation in anaesthetic and perioperative techniques across the UK, although the dissemination of Enhanced Recovery principles has increased consistency of perioperative practice between and within units. As ALLEGRO is a trial of a perioperative adjunct to normal care, we judged it impracticable to enforce a single strict anaesthetic/perioperative protocol. If found to be effective in this setting, it would provide evidence of external validity/applicability suggesting it could also be a useful adjunct in other gastrointestinal/abdominal surgery.

The majority of elective colorectal surgery in the UK is now undertaken using minimally invasive techniques (laparoscopic or robotic), but there remains a role for open surgery in some cases (dictated by various factors including case selection and surgeon preference). Initially, ALLEGRO included a separate exploratory study, with separate randomisation, for planned open cases. This study was deliberately exploratory and contained no estimate of sample size, as we were uncertain as to the rate of elective open surgery in recruiting centres and whether clinicians would accept equipoise given that many anaesthetists prefer epidural anaesthesia for these cases. By early 2020, only 12 participants had been recruited to the exploratory study for open cases (most in the CIs site), in comparison to 201 participants in the main laparoscopic trial, suggesting an absence of equipoise. With approval from the funder, the exploratory study for planned open cases was closed as part of protocol version 5 (8 September 2020), by which time 16 participants had been recruited into the exploratory study.

In common with many studies, recruitment to ALLEGRO was halted by the COVID pandemic. However, the essential nature of the surgery (predominantly for cancer) meant that eligible cases restarted relatively quickly, and the original design of the study, which had aimed to integrate trial processes with existing hospital contact points in the surgical patient pathway (outpatient clinics, preassessment appointments) meant that no change of trial design was required to meet COVID restrictions. However, we did alter some processes around consent and giving patient information to allow research teams to have a consent discussion by telephone with participants subsequently returning the completed consent form by post.

The need to randomise participants before date of operation in order to have the trial drug dispensed from clinical trial pharmacies ready for the start of surgery has resulted in unanticipated issues in some cases resulting in a relatively high number of post-randomisation exclusions. The main reasons for these are change in planned type of surgery (particularly open rather than minimally invasive technique; a preoperative decision to make a new stoma) or change in anaesthetic technique (particularly decision to use an epidural) made on the morning of surgery. This is a challenging area for trial teams: it can be difficult to know which surgical/anaesthetic staff are undertaking an operating list until relatively late, there may be nuances about the planned operation known only to the operating surgeon and not always stated on the operating theatre list and there is varying integration of research nurses within the participating surgical/anaesthetic teams. At the current rate, we anticipate approximately 20–25 post randomisation exclusions; the appropriate number of cases will be added to the study to achieve the target sample size of 562 cases.

## Trial status

Protocol version 5; 08 September 2020

Start recruitment: 14 August 2018

Estimated completion of recruitment: 31 December 2022

## Abbreviations

ACCORD: Academic and Clinical Central Office for Research & Development - Joint office for The University of Edinburgh and Lothian Health Board
AE: Adverse event
AES: Advanced Encryption Standard
ACPGBI: Association of Coloproctology of Great Britain and Ireland
BDRF: Bowel Disease Research Foundation
CI: Chief investigator
CRF: Case report form
CRP: C-reactive protein
DMC: Data monitoring committee
eGFR: Estimated glomerular filtration rate
EQ-5D-5L: European Quality of Life- 5 Dimensions
ERAS: Enhanced Recovery After Surgery
EudraCT: European Clinical Trials Database
GCP: Good Clinical Practice
GI: Gastrointestinal
GP: General practitioner
HCRU: Health care resource utilisation
IMP: Investigational medicinal product
ISRCTN: International Standard Randomised Controlled Trials Number
IV: Intravenous
MHRA: Medicines and Healthcare products Regulatory Agency
NaCl: Sodium chloride
NHS: National Health Service
NHS R&D: National Health Service Research & Development
NICE: National Institute for Health & Care Excellence
OBAS: Overall Benefit of Analgesia Score
PMG: Project management group
POMCTN: UK Perioperative Medicine Clinical Trials Network
PONV: Postoperative nausea and vomiting
PPOI: Prolonged postoperative ileus
p-POSSUM: Physiological and Operative Severity Score for the enUmeration of Mortality and Morbidity.
PROM: Patient-reported outcome measurement
PSA: Probabilistic sensitivity analysis
PSS: Personal social service
QALY: Quality-adjusted life year
QoR: Quality of recovery
RCT: Randomised control trial
REC: Research Ethics Committee
SAE: Serious adverse event
SAP: Statistical analysis plan
SAR: Serious adverse reaction
SSL: Secure sockets layer
SUSAR: Suspected unexpected serious adverse reaction
TSC: Trial steering committee
UK: United Kingdom
